# LoVis4u: a locus visualization tool for comparative genomics and coverage profiles

**DOI:** 10.1093/nargab/lqaf009

**Published:** 2025-02-24

**Authors:** Artyom A Egorov, Gemma C Atkinson

**Affiliations:** Department of Experimental Medical Science, Lund University, 221 84, Lund, Sweden; Department of Experimental Medical Science, Lund University, 221 84, Lund, Sweden

## Abstract

Comparative genomic analysis often involves visualization of alignments of genomic loci. While several software tools are available for this task, ranging from Python and R libraries to stand-alone graphical user interfaces, a tool is lacking that offers fast, automated usage and the production of publication-ready vector images. Here we present LoVis4u, a command-line tool and Python API designed for highly customizable and fast visualization of multiple genomic loci. LoVis4u generates vector images in PDF format based on annotation data from GenBank or GFF files. It is capable of visualizing entire genomes of bacteriophages as well as plasmids and user-defined regions of longer prokaryotic genomes. Additionally, LoVis4u offers optional data processing steps to identify and highlight accessory and core genes in input sequences. Finally, LoVis4u supports the visualization of genomic signal track profiles from sequencing experiments. LoVis4u is implemented in Python3 and runs on Linux and MacOS. The command-line interface covers most practical use cases, while the provided Python API allows usage within a Python program, integration into external tools, and additional customization. The source code is available at the GitHub page: github.com/art-egorov/lovis4u. Detailed documentation that includes an example-driven guide is available from the software home page: art-egorov.github.io/lovis4u.

## Introduction

The exponential growth of microbial genome databases has unlocked numerous opportunities for comparative genomic analyses [1]. Various tasks such as analysis of gene neighbourhood conservation [2, 3], annotation of functional short open reading frames (ORFs) [4, 5], and investigation of genomic variability hotspots [6–8] often require visualization of multiple genomic loci. Several software tools have been developed for this purpose. A subset of these have graphical user interfaces (GUIs), such as the Artemis Comparison Tool [9], Easyfig [10], GeneSpy [11], and Geneious Prime (geneious.com). Another category comprises web-based applications like Gene Graphics [12]. Additionally, there are libraries such as the R packages genoPlotR [13] and gggenes [14], as well as the Python package GenomeDiagram [15]. Some tools integrate multiple approaches, creating hybrid solutions. For example, GEnView is a Python pipeline combined with an interactive web application [16], and Clinker & clustermap.js [17] is a popular tool with a command-line interface and an interactive web application that can generate vector graphics. While many of these tools feature interactivity through GUIs or web applications, there is a lack of a user-friendly command-line tool suitable for handling multiple input genomes, containing data analysis steps, flexible customization options, and with fast production of aesthetically pleasing publication-ready figures.

Here we present LoVis4u (Locus Visualization), a scalable software tool designed for customizable and fast visualization of multiple genomic loci. LoVis4u offers a command-line interface without requiring user-side scripting and provides a Python API for additional customization and integration within Python programs. In addition to visualization features, our tool has optional data analysis steps that include protein clustering to find groups of protein homologues with subsequent identification of accessory and core genes that can be highlighted with visualization. In addition, hidden Markov model searches enable fast functional annotation of proteins using a broad set of model databases. At the nucleotide level, LoVis4u can visualize sequencing track data such as GC content and GC skew, along with next-generation sequencing coverage profiles. While LoVis4u was designed for annotating and visualizing multiple bacteriophage genomes or their loci, it can also be used for visualizing defined regions of any prokaryotic genome.

## Materials and methods

### Design and implementation

**Figure 1.**
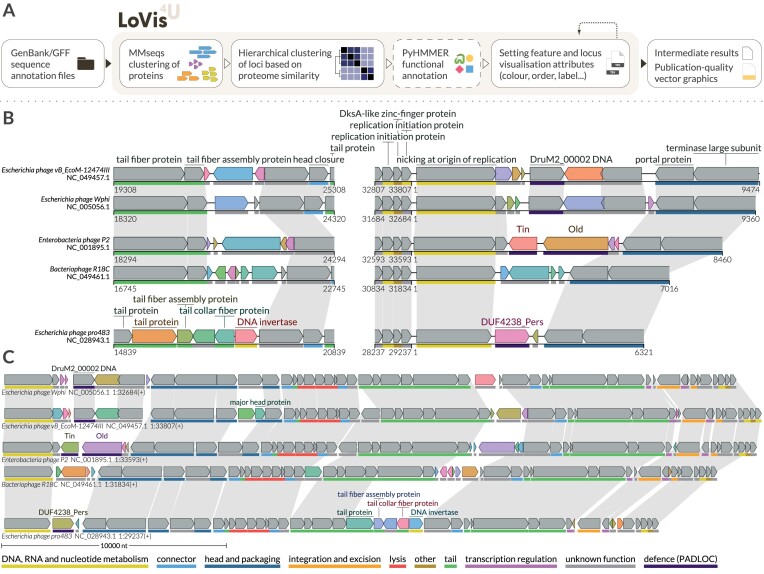
LoVis4u workflow and visualization example. (**A**) Schematic description of the LoVis4u pipeline and its input and output. (**B**) Visualization of multiple regions for a set of P2-like phage genomes. Conserved genes are shown in grey, while variable protein groups are highlighted with distinct colours. Homologous protein groups defined by MMSeqs2 clustering and located on different genomes are connected by grey homology lines. As can be set in the configuration file, protein labels are hidden for hypothetical proteins or proteins of unknown function. Labels for conserved proteins are shown only for the first occurrence since homology lines indicate additional occurrences. Label positions are arranged automatically by the algorithm to avoid overlaps of text. PHROG and PyHMMER functional annotations [38] are indicated by coloured lines beneath each ORF, according to the colour code at the bottom of the panel. (**C**) Visualization of a set of P2-like phage genomes that showcases a compact visualization of full-length sequences, where functional annotation tracks and individual *x*-axes are hidden. Instead, a scale line at the bottom is displayed to indicate region size.

The LoVis4u pipeline includes several default data processing steps, which are optional depending on user needs (Fig. 1A). LoVis4u supports input data in either GenBank or extended GFF (concatenated with the corresponding nucleotide sequence in fasta format) file format. GFF files in this format are produced by the widely used prokka [18] and pharokka [19] genome annotation tools.

By default, LoVis4u applies the MMseqs2 [20] protein clustering algorithm to all encoded protein sequences to identify groups of homologous proteins. Alternatively, a table with predefined protein groups can be used as input. Based on the defined protein groups, LoVis4u constructs a matrix of pairwise proteome composition similarity scores that reflect the fraction of shared homologous proteins between sequences, and a corresponding proteome composition distance matrix. The clusters and order of input sequences for visualization are determined using hierarchical clustering with average linkage applied to the distance matrix. This approach allows us to consider a set of genes for each cluster analogously to pangenome analyses, where we define ‘conserved’, ‘intermediate’, and ‘variable’ protein group classes that are roughly equivalent to ‘core’, ‘shell’, and ‘cloud’ terms of pangenomics [21]. Finally, the optional PyHMMER [22, 23] hmmscan search step for protein functional annotation can be applied. The list of available databases for search includes DefenseFinder and CasFinder (defence) [24, 25], PADLOC (defence) [26], dbAPIS_Acr (anti-defence) [27], VFDB (virulence factors) [28], and AMRFinderPlus (AMR genes) [29]. LoVis4u can read genomic signal data in bedGraph and bigWig file formats [30]. A detailed workflow description is available on the LoVis4u home page (art-egorov.github.io/lovis4u).

LoVis4u can be run in quick-start mode, with few required options, but also has a range of advanced customization options to give users full control over the output. Sequence order, sequence clusters, and protein group classes can be manually specified through feature and locus annotation tables, which can be provided as optional arguments. LoVis4u has a set of editable configuration files with advanced parameters already adjusted for different tasks and page layouts, including one- and two-column A4 page layouts. Users can also customize colours and labels with these files. A step-by-step guide on the home page demonstrates how to experiment with and optimize visualizations using these features. Finally, LoVis4u uses the ReportLab library API to generate the output vector image, which is saved in PDF format. This format allows further editing of all objects in vector image-editing programs like Adobe Illustrator or Inkscape if needed.

LoVis4u is implemented in Python3 and uses multiple Python3 libraries: biopython [31], bcbio-gff, scipy [32], configs, argparse, pandas [33], distinctipy [34], matplotlib [35], seaborn [36], reportlab, pyhmmer [22, 23], progress, and requests. LoVis4u also uses MMseqs2 [20] and bigWigToBedGraph [30] as non-Python dependencies, which are embedded in the library.

## Results

### Demonstration of LoVis4u key features in comparative genomics

To test and demonstrate a subset of the tool’s key features, we visualized a set of P2-like phage genomes obtained from the RefSeq database [37] (Fig. 1B and C). LoVis4u allows users to specify multiple regions for each sequence, which are displayed on a single line. Additionally, if a sequence is circular (as is the case with many phage and plasmid sequences), the visualization will continue without a gap between sequence end and start coordinates. By default, LoVis4u highlights ‘variable’ proteins by automatically assigning different colours to each homologous protein group. Alternatively, conserved proteins can be highlighted. LoVis4u also automatically interprets PHROG [38] functional group annotations, as provided in GFF and GenBank files produced by Pharokka together with results of PyHMMER search, and can display these annotations with a functional category line beneath the ORFs. To better visualize long sequences, LoVis4u includes options for a more minimalistic and compact design as shown in panel Fig. 1C. A step-by-step guide for creating these figures can be found in the user guide on the tool’s home page together with the gallery of more examples and command-line options to reproduce them: art-egorov.github.io/lovis4u/Gallery/gallery.

The capabilities and potential of the LoVis4u API was recently demonstrated in the systematic annotation of variable hotspots in phage genomes and plasmid sequences, where LoVis4u was integrated for high-throughput and fast visualization of millions of loci [39].

### Exploration of the BASEL collection of phages with LoVis4u

**Figure 2.**
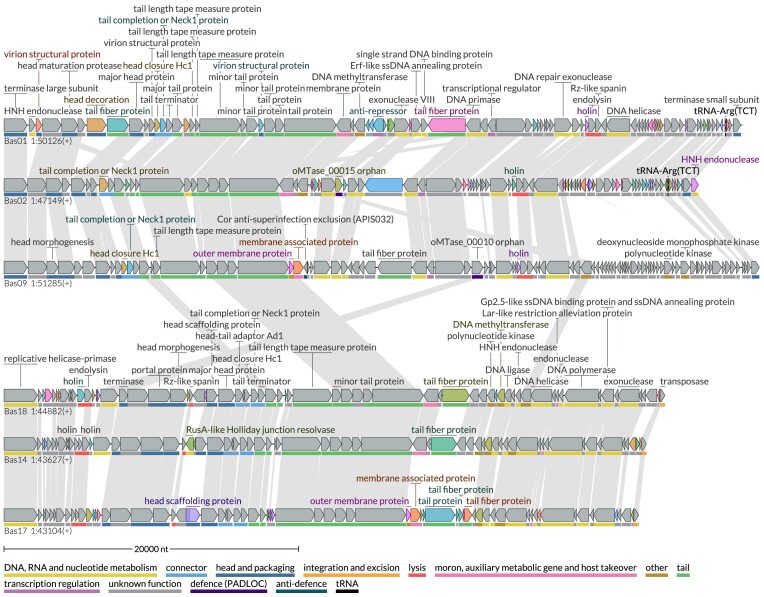
LoVis4u identifies regions of homology within the BASEL phage collection. Visualization of a set of BASEL [40] phage genomes. Colours and annotations are as per Fig. 1.

To demonstrate full-length sequence visualization of a larger set of sequences, we used LoVis4u to create an overview of the complete BASEL collection of 78 phages [40] in a single figure, highlighting the variable genes (Supplementary File S1). The pipeline, which includes clustering of 13 630 protein sequences, hierarchical clustering of sequences, and generating the final graphic output, completed in only 50 seconds on an M1 MacBook Pro laptop. Beyond visualization, a key feature of LoVis4u is its ability to identify clusters of similar proteomes within the dataset and distinguish accessory and core proteins within each cluster. LoVis4u also generates a pairwise proteome similarity matrix, visualized for the BASEL collection in Supplementary Fig. S1. The dendrogram produced through clustering has been used to illustrate results from a phage screening experiment [41, 42]. Visualization of a subset of six BASEL phages is presented in Fig. 2, highlighting an additional capability of LoVis4u: its ability to process non-coding features, such as tRNAs.

### Genome browser mode

In addition to comparative analysis and visualization of multiple related loci, another common task in microbiology is the analysis of a single genome and the visualization of coverage profiles from sequencing experiments (e.g. DNA-seq or RNA-seq), either for an entire genome or for user-defined windows. To address this, LoVis4u includes a genome browser mode specifically designed for prokaryotic genomes. In addition to visualization of multiple genomic signal tracks from sequencing experiments (e.g. DNA-seq, RNA-seq), it can generate output figures with sequence property tracks, such as GC content and GC skew profiles. LoVis4u also automatically applies moving-average smoothing to signal tracks when needed, taking into account the image width and the size of the visualized window.

**Figure 3.**
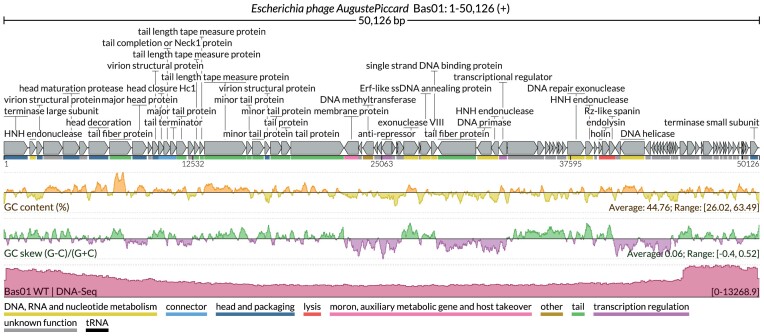
LoVis4u genome browser mode. Visualization of the *Bas01 Escherichia* phage Auguste Piccard genome. The functional annotation track and gene map are as shown in Fig. 1. GC content and GC skew tracks are centred at average values, with positive and negative relative values represented by different colours. The range and average values are annotated in the bottom right corner of each track. The genomic signal track is shown in the lowest track, in dark red. Multiple additional signal tracks, e.g. to compare wild type and mutants, can be added below.

To demonstrate this mode, we applied LoVis4u to Bas01 *Escherichia*phage Auguste Piccard from the BASEL collection [40], using a genomic signal track obtained from an example DNA-seq experiment (data distributed with the package sample data) (Fig. 3).

## Discussion

Comparative genomic analysis in microbiological studies often requires specialized visualization tools for various tasks. Here, we present LoVis4u, a software tool designed to produce publication-quality figures of genomic loci with several automated analysis steps integrated into the pipeline. LoVis4u offers a much-needed compromise between tools with advanced R/Python APIs and those with user-friendly graphical interfaces and interactivity. It can be used for fast and automated generation of multiple figures in bioinformatics pipelines or other libraries, or can be used as a stand-alone tool for data exploration. By sensitively finding and annotating conserved and variable regions, LoVis4u can facilitate comparative evolutionary analyses of genomes or genomic regions, and the discovery of new biology. Finally, the LoVis4u genome browser mode provides a powerful tool for exploring results from sequencing experiments, such as DNA-seq or RNA-seq, facilitating both analysis and visualization to address different microbiological problems.

## Supplementary Material

lqaf009_Supplemental_Files

## Data Availability

The Python LoVis4u package is available in PyPI (*python3 -m pip install lovis4u*), and the source code is provided on GitHub (github.com/art-egorov/lovis4u) and Zenodo (https://doi.org/10.5281/zenodo.14800717). Detailed documentation with an installation guide and an example-driven manual are available on the LoVis4u home page (art-egorov.github.io/lovis4u).
