# Supplementary material for: LoVis4u: a locus visualization tool for comparative genomics and coverage profiles
Source: NAR Genom Bioinform. 2025 Feb 24;7(1):lqaf009. doi: 10.1093/nargab/lqaf009 (PMC11850299; doi:10.1093/nargab/lqaf009)
Supplement: lqaf009_Supplemental_Files [file lqaf009_supplemental_files.zip › SupplemetaryInformation.pdf]

Supplementary Information

LoVis4u: a locus visualisation tool for comparative genomics

Artyom A. Egorov[1#], Gemma C. Atkinson [1#]

[1] Department of Experimental Medical Science, Lund University, Lund, Sweden

[#] To whom correspondence should be addressed:

Artyom A. Egorov, artem.egorov@med.lu.se

Gemma C. Atkinson, gemma.atkinson@med.lu.se

Supplementary Figures

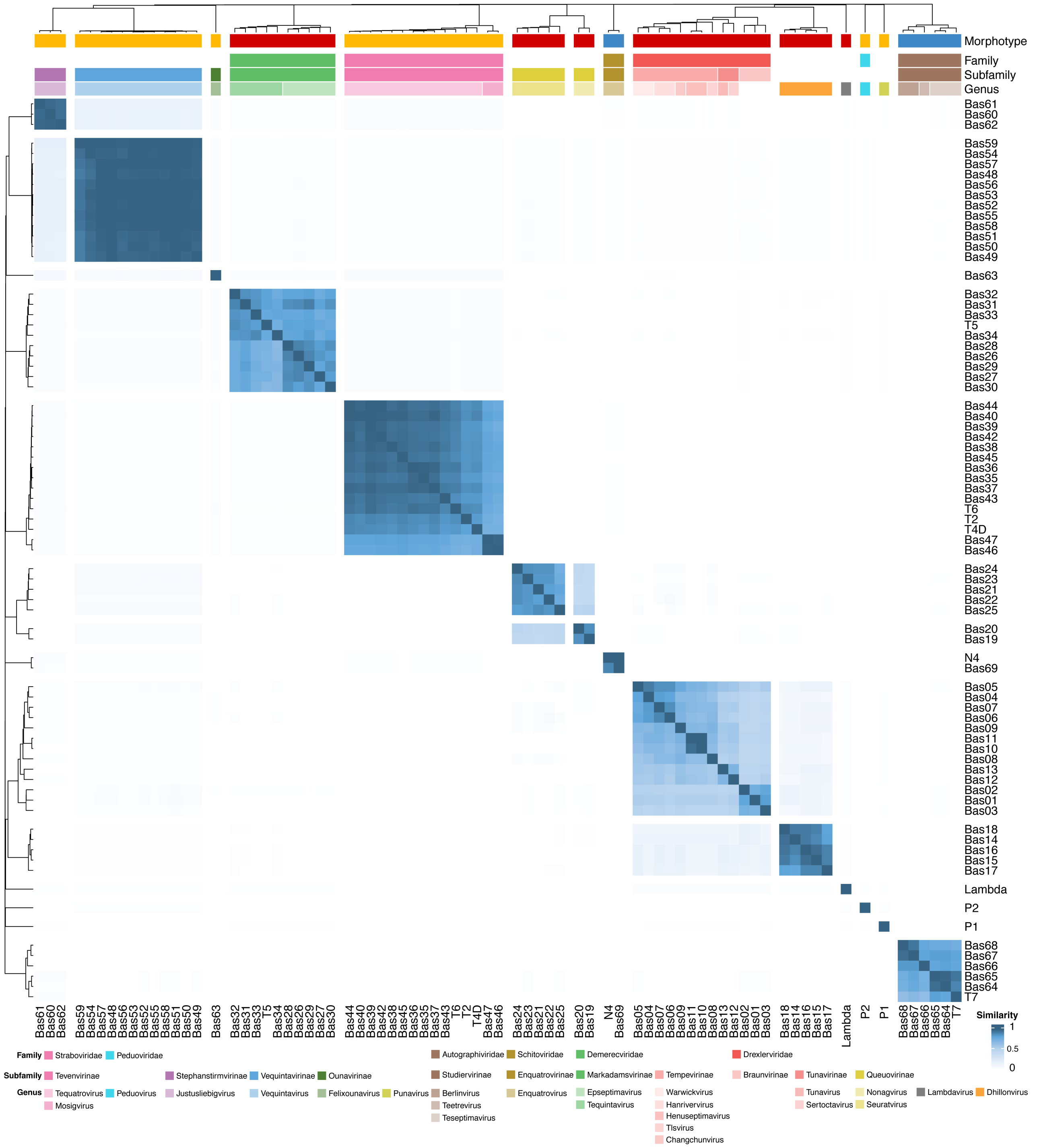

Supplementary figure S1 | Proteome similarity matrix of the BASEL collection of phages. LoVis4u calculates symmetric pairwise similarity scores, *sim(i,j)*, reflecting the fraction of shared homologous proteins between two proteomes. These scores are then used to build the

proteome composition distance (PCD) matrix, with distances defined as $dist(i,j) = 1 - sim(i,j)$. Hierarchical clustering with the average-linkage method is applied to the corresponding PCD matrix, and the resulting dendrogram determines the order of sequences in the visualisation.
