# Supplementary figures and images for: LoVis4u: a locus visualization tool for comparative genomics and coverage profiles
Source: NAR Genom Bioinform. 2025 Feb 24;7(1):lqaf009. doi: 10.1093/nargab/lqaf009 (PMC11850299; doi:10.1093/nargab/lqaf009)

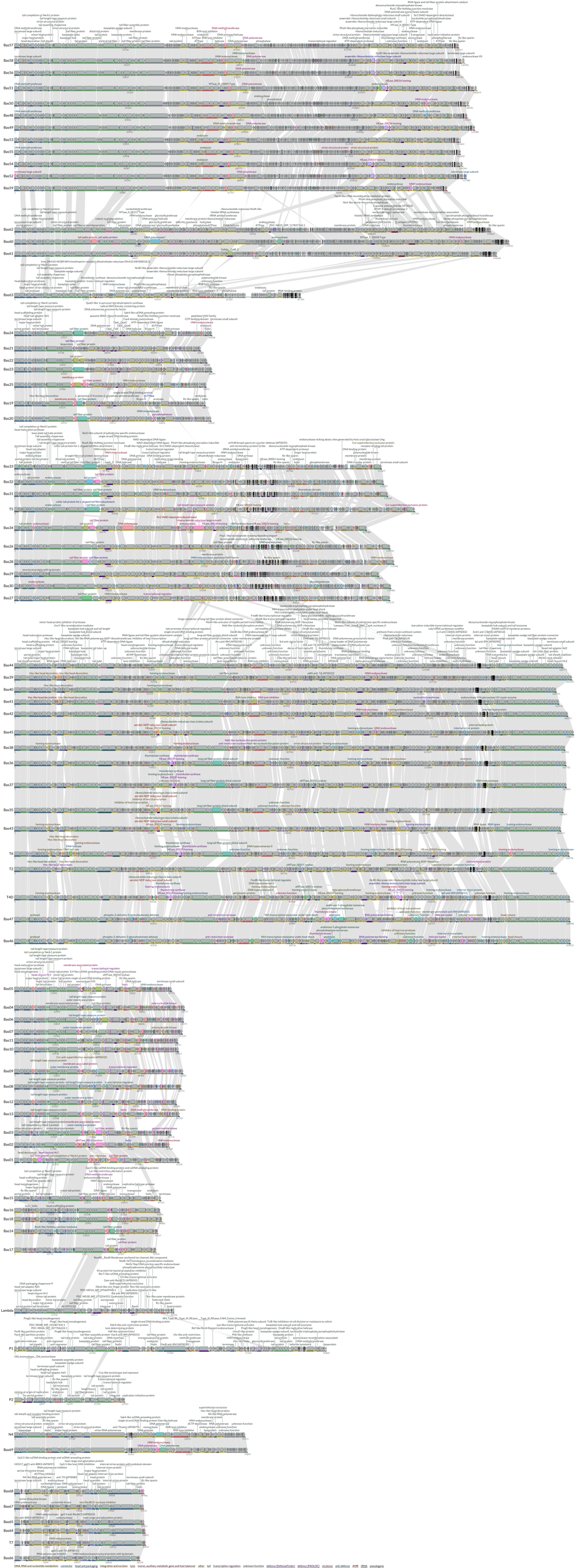

Supplement: lqaf009_Supplemental_Files [file lqaf009_supplemental_files.zip › SupplementaryFile1.pdf]
